# Sepsis-associated encephalopathy: Mechanisms, Diagnosis, and Treatments update

**DOI:** 10.7150/ijbs.102234

**Published:** 2025-04-28

**Authors:** Jin Li, Qi Jia, Lin Yang, You Wu, Yuliang Peng, Lixia Du, Zongping Fang, Xijing Zhang

**Affiliations:** 1 Department of Critical Care Medicine, Xijing Hospital, Fourth Military Medical University, Xi'an 710032, Shaanxi, China.; 2 Department of Anaesthesiology and Perioperative Medicine,Xijing Hospital, Fourth Military Medical University, Xi'an 710032, Shaanxi, China.; 3 Department of Critical Care Medicine, Fourth People's Hospital, School of Medicine, Tongji University, Shanghai 200434, China.; 4 Department of Critical Care Medicine, Air Force Medical Center, Beijing 100142, China.

**Keywords:** sepsis-associated encephalopathy, delirium, neuroinflammation, diagnosis, treatment

## Abstract

Sepsis-associated encephalopathy (SAE) is defined as a syndrome of cerebral dysfunction secondary to sepsis but in the absence of direct central nervous system infection, structural abnormality, or other types of encephalopathy. The majority of clinical studies indicated that the severity and duration of SAE were highly related to the days of ICU stays, medical costs, and mortality of sepsis. Meanwhile, the persistence of cognitive impairments and psychological diseases in a majority of survived septic patients brings a heavy burden on those individuals and society. However, the pathogenesis of SAE has not been fully elucidated. A valid and unified diagnosis protocol, as well as effective remedy are still absent. The purpose of this narrative review is to discuss and update the current understanding of the clinical manifestations and risk factors, the recent findings and potential perspectives for the mechanism research, diagnostic methods, and treatments for SAE.

## Introduction

The current definition of sepsis was established by the Third International Consensus on Sepsis and Septic Shock in 2016, known as the “sepsis-3” criterion^1^. In this criterion, sepsis is defined as life-threatening organ dysfunction caused by a dysregulated host response to infection. An estimated 48.9 million patients suffered sepsis worldwide in 2017 and 11.0 million of them eventually died^2^. Of all the sepsis patients, more than half of them need treatment in intensive care unit (ICU).

As a major complication secondary to sepsis, SAE is manifested as a spectrum of disturbed cerebral function, ranging from mild delirium to coma^3^. A large sample retrospective analysis of a multicenter database showed that 53% (1341/2351) of the patients who suffered sepsis presented delirium and coma at the time of ICU admission^4^. This study also suggested that older patients with a history of chronic alcohol abuse, neurological disease, pre-existing cognitive impairment, and long-term use of psychoactive drugs might be more susceptible to SAE. Furthermore, complications including acute renal failure, metabolic disturbances, dysglycemia, hypercapnia, and hypernatremia could perform as risk factors in contributing to the incidence of SAE. Overall, it seems that sepsis patients with disturbed cerebral function tended to have a heavier burden of systemic illness and were associated with higher mortality. However, should those systemic illnesses and disturbances be considered to be confounders or diagnostic indicators for SAE are debatable.

## Mechanisms and potential therapeutic targets of SAE

**Figure 1:**
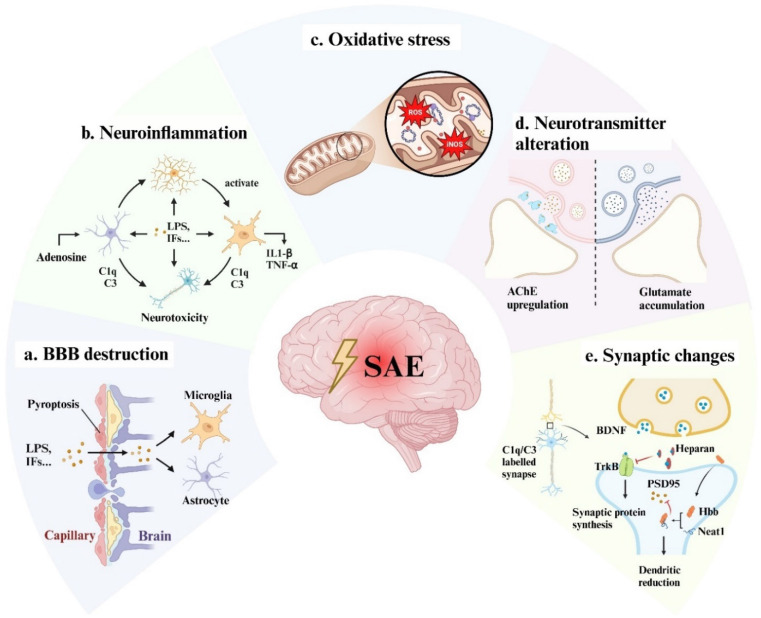
** The possible mechanisms of SAE.** Bacterial toxic components such as LPS and peripherally produced inflammatory factors act on vascular endothelial cells causing pyroptosis, cell edema, cell junction dysfunction, and vascular permeability alteration, resulting in a large number of inflammatory factors passing through the blood-brain barrier (a). LPS and inflammatory cytokines activate microglia and astrocytes to release pro-inflammatory factors and complement to produce neurotoxicity (b) and aggravate BBB disruption. At the same time, those factors can also directly act on neuronal cells to cause neuronal death (b). Intracellularly, in response to inflammation and hypoxia, mitochondria produce a large amount of ROS and iNOS, which cause oxidative stress and ultimately lead to energy supply disorders (c). The functional alterations of neurons and glial cells may also lead to neurotransmitter (d) and synaptic plasticity disorder (e), which are responsible for the long-term cognitive impairment of SAE patients. LPS: lipopolysaccharide; IFs: inflammation factors; TJs: tight junctions; ECs: endothelial cells; ROS: reactive oxygen species; iNOS: inducible nitric oxide synthase; AChE: acetylcholinesterase; BDNF: brain-derived neurotrophic factor; Neat1: lncRNA nuclear enriched abundant transcript 1; PSD-95: post-synaptic density protein 95.

It is widely accepted that patients with septic shock are more likely to suffer cerebral dysfunction^5^. Hypoperfusion, hypoxia, microthrombosis, and internal environment disturbance are thought to be the leading causes of multi-organ dysfunction including the brain. Guidelines such as the Surviving Sepsis Campaign recommended early goal-directed therapy and organ replacement therapy to reverse shock and protect organs^6^. However, considering that the brain is vulnerable and unreplaceable (in compare with kidney and liver), specific neuroprotective interventions are urgently required. Therefore, we discussed the mechanisms of SAE at the cellular and molecular level (Fig 1), which might provide new insight into SAE therapy.

### Blood-Brain Barrier (BBB) disruption

The BBB is a tightly sealed selectively permeable membrane between the peripheral circulation and the brain parenchymal cells. Its integrity and function are essential for maintaining the stability of the internal environment of brain tissue and the normal function of brain cells. At the molecular level, the BBB endothelial cells (ECs) are sealed by tight junctions (TJs). Autopsy series of fatal sepsis patients have illustrated that TJ proteins are significantly down-regulated, indicating that damaged BBB is related to severe sepsis^7^. The potential mechanisms and therapeutic targets for BBB disruption in sepsis or SAE have been studied for years.

Pattern recognition receptors known as Toll-Like Receptors (TLRs) are ubiquitously expressed and localized on the cell membrane surface where they function as essential mediators in responding to pathogens and inflammation signals. The binding of lipopolysaccharide (LPS) with TLR-4/myeloid differentiation 2 (MD-2) complex on cerebral ECs membrane has been proven to activate the nuclear factor-κ-gene binding (NF-κB) signaling pathway, which mediates intracellular oxidative stress response, causing ECs edema and TJs disassembly^8^. Although it has been suggested that LPS stimulates TLR-4 to produce inflammatory factors that aggravate BBB damage, the most recent study showed that exogenous administration of TNF-α and IL-6 did not cause damage to the blood-brain barrier^9^. In addition, this study found that LPS-induced BBB damage could be independent from TLR-4. LPS endocytosis mediated by LPS binding protein (LBP)/CD14 could activate the intracellular receptor caspase11/4 and induce gasdermin-D (GSDMD)-mediated plasma membrane permeability increase and pyrosis. And the administration of an inhibitory nanoparticle targeting the human GSDMD-N domain could effectively block the LPS-induced blood-brain barrier destruction^9^.

Some studies have attempted to improve SAE by repairing the BBB. The nuclear factor erythroid 2-related factor 2 (Nrf-2) is one of the major regulators of cellular endogenous antioxidant systems and is related to a variety of neurodegenerative diseases^10^. Targeting on NF-κB or/and Nrf-2 signaling pathways have been proven to be effective in restoring BBB permeability in numerous studies^11^-^15^. However, the interventions involved in those studies have not been validated by well-designed clinical trials yet. In addition, matrix metalloproteinase (MMP), secreted by the ECs, is also involved in the process of BBB dysfunction, especially MMP-2 and MMP-9. Regulated by cytokines of the mitogen-activated protein kinase family pathway, MMP-2 and MMP-9 cause the basement membrane and TJs degradation, thereby elevating BBB permeability^16^. A recent study observed the protective effects of high-dose vitamin C against MMP-9 upregulation, and subsequently attenuated BBB disruption and cognitive impairment in SAE mice^17^. But regrettably, the latest clinical trial reported that high-dose intravenous vitamin C might be harmful in patients with severe sepsis^18^. Therefore, the use of vitamin C in the treatment of SAE or related clinical studies may be ethically challenged, unless safe targeted drug delivery methods are employed. In summary, anti-inflammatory therapy may not be sufficient to prevent disruption to the BBB caused by sepsis, and anti-pyroptosis or anti-oxidative stress therapies targeting ECs may be of potential for further research.

### Neuroinflammation and glial cells activation

Neuroinflammation is defined as inflammation within the central nervous system (CNS) characterized by the activation of neuroglial cells (mostly microglia and astrocytes) and increased inflammatory mediators in the cerebral parenchyma^19^. Under non-septic infectious conditions, pro-inflammatory factors such as IL-6 and TNF-α can enter the cerebrospinal fluid through transporters to assist the central nervous system in sensing peripheral inflammatory responses and modulating peripheral inflammation via the neuro-immune axis. However, once the infection progresses to sepsis, a compromised BBB leads to dysregulation and a lack of selectivity within this process^20^. Single-nucleus RNA sequencing and spatial transcriptomics data from SAE mice illustrated that excessive pro-inflammatory factors entering the CNS activate glial cells, triggering severe neuroinflammatory responses^21^.

Microglia are the main innate immune cells of the CNS. Similar to peripheral macrophages, microglia can be activated into M1 and M2 subtypes, representing pro- and anti- inflammation respectively. Under inflammation state, inhibiting M1 or promoting M2 polarization can alleviate neuroinflammation and cognitive dysfunction in sepsis mice^22^, ^23^. The role of microglia in neuroinflammation mainly depends on membrane pattern recognition receptors (PRRs) for recognizing different pathogen-associated molecular patterns (PAMPs) upregulated during sepsis^24^. Among various PRRs, TLRs play unique roles in innate immune responses to sepsis. As mentioned above, TLR-2 and TLR-4 are considered to be key PAMPs receptors in neuroinflammation^25^. Activation of TLRs triggers the cellular pathways responsible for the nuclear localization of NF-κB, leading to the assembly of the inflammasome such as NOD-, LRR- and pyrin domain-containing protein 3 (NLRP3) who cleaves pro-IL-1β and GSDMD. Cleaved N-terminal GSDMD inserts into the membrane, forming pores and inducing IL-1β releasing and pyroptosis^26^. IL-1β-induced hippocampal neuronal dysfunction in SAE mice has been demonstrated^27^, ^28^, and a recent study revealed that extracellular vesicles released from pyrocytes can transplant GSDMD pores to the surface of bystander cells propagating pyroptosis^29^. Although the role of this mechanism in microglia and SAE has not been validated, it may explain the widespread immune cell death and tissue (including brain) damage in sepsis.

Astrocytes are the homeostatic cells of the CNS with a wide array of functions such as lipid metabolism, neurotransmitter reuptake, and synaptic plasticity maintenance^30^. Its role in neuroinflammation is less important than that of microglia. Previous views suggested that the inflammatory factors released by microglia could activate astrocytes, and reactive astrocytes could release complement C3 to mediate neurotoxicity^31^, ^32^. Recent study has shown that the adenosine, increased in the plasma during sepsis, activates astrocytes within the first 6 h after LPS challenge and further activates microglia^33^. Although the specific ablation of adenosine receptor in astrocytes did not prevent the occurrence of SAE, it still delayed microglia activation and reduced the level of inflammatory factors in the brain^33^. This is perhaps the most important aspect of SAE that distinguishes it from other neuroinflammatory or neurodegenerative diseases, namely that SAE is secondary to a violent peripheral inflammatory response. Therefore, small molecules elevated in the peripheral plasma may play an important role in the pathogenesis of neuroinflammation. Clearance of these substances by certain means such as continuous renal replacement therapy may be helpful for the treatment of SAE.

**Figure 2:**
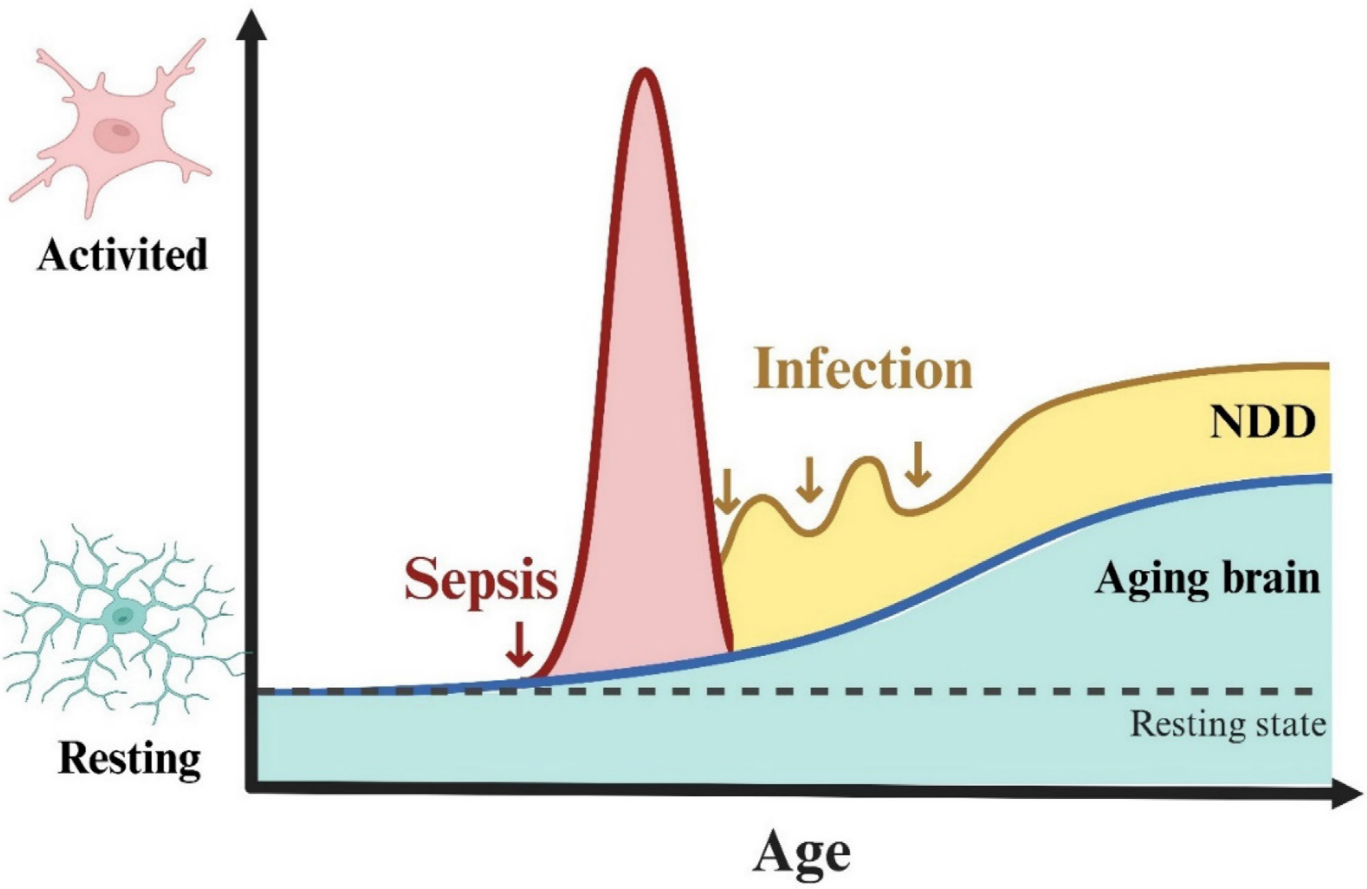
** Speculation on the correlation between sepsis and neurodegenerative diseases.** Aging and neurodegenerative diseases are strongly associated with chronic activation of microglia (also known as disease-associated microglia). Based on the association of sepsis with the risk of neurodegenerative diseases, we hypothesized that sepsis causes acute inflammatory activation of microglia, but this activation is restored within a relatively short period of time. In the restoring phase, repeated infections (although not severe enough to cause sepsis) can also cause microglia to develop immune memory, chronic inflammatory activation, and eventually differentiation into disease-associated microglia leading to neurodegenerative disease**.** NDD: neurodegenerative diseases.

Cell death or dysfunction caused by acute neuroinflammation is an important mechanism of SAE pathogenesis. However, the relationship between chronic inflammation after sepsis and SAE is poorly understood. An important recent study found that IL-1β can mediate the process of innate immune memory in bone marrow hematopoietic stem cells^34^, which leads to chronic inflammation after sepsis. Immune memory in microglia was also observed in the mouse brain, where a single intraperitoneal injection of LPS induced immune training and resulted in differential epigenetic reprogramming of microglia for at least 6 months^35^. We do not yet know how long the innate immune memory of human microglia will maintain, but clinical data suggests that SAE patients suffer long-term cognitive impairment lasting up to 2 years after sepsis^36^, ^37^, and SAE patients are at significantly increased risk of developing neurodegenerative diseases such as Alzheimer's disease^26^. We speculate that this may be related to the long-term activation of microglia induced by multiple relatively mild infections after sepsis (Fig 2). Research in this area may have significant implications for the out-of-hospital management of sepsis survivors.

### Mitochondria dysfunction and oxidative stress

Due to the high oxygen consumption and the decrease in oxygen supplements, the brain is more prone to suffering hypoxia and much more vulnerable in sepsis and sepsis shock. The electron transport chain (ETC) inhibition and mitochondria membrane disruption secondary to severe inflammation are proposed to be the mechanism of mitochondria dysfunction^38^. The disruption of ETC resulted in a blockage of electron transport, and the electron extravasation led to the production of reactive oxygen species (ROS) at the sites of complex Ⅰ and Ⅲ^26^. On the other hand, inducible nitric oxide synthase (iNOS) elevated in the brain as a proinflammation factor soon as the onset of sepsis^39^. ROS and NO, as the main executors of oxidative stress, subsequently triggered a series of intracellular reactions such as protein misfolding and lipid peroxidation once they exceeded the upper loading limit of the antioxidant system^40^. The accumulation of oxygen radicals is also involved in the activation of signaling pathways related to neuroinflammation and cell death^41^, ^42^, part of which we have discussed in the section of neuroinflammation. And studies have shown that inhaling hydrogen or using hydrogen-rich saline can improve SAE symptoms by scavenging free radicals^43^, ^44^. Of note, ferroptosis, a novel form of cell death, is closely associated with ROS and lipid peroxidation. Evidence suggests that during SAE, microglia undergo ferroptosis and exacerbate neuroinflammation^41^. However, there is currently no compelling evidence of ferroptosis occurring in neurons in either patients or sepsis animal models.

In addition to initiating oxidative stress, mitochondria play critical roles in calcium homeostasis, vesicular cycling, and membrane ion channel activity in neurons. These functions are essential for synaptic communication within the nervous system, particularly for presynaptic neurotransmitter release^45^. It has been proved that presynaptic neurotransmitter release is significantly inhibited in SAE as evidenced by the frequency of postsynaptic conductance data from various studies and models^46^-^49^. This impairment may be attributed to mitochondrial dysfunction, resulting in ATP deficiency and the inhibition of active ion transport. Damaged mitochondria should be cleared through mitophagy, which prevents the release of mitochondrial damage-associated molecular patterns (DAMPs), such as mtDNA^50^. However, mitochondrial damage may exceed the clearance capacity of mitophagy during SAE. Studies have demonstrated that pharmacological enhancement of mitophagy can improve SAE in animal models^51^, ^52^. While enhancing mitophagy may alleviate neuroinflammation, it does not theoretically address the underlying issue of cellular energy deficiency. A recent study indicated that astrocytes can transfer mitochondria to neurons mediated by ADP-ribosylation factor 1 (ARF1), thereby increasing the tolerance capability of neurons to hypoxia^53^, ^54^. However, hyperlactatemia induced by sepsis may impair this process by promoting ARF1 lactylation^54^. Therefore, clinical trials investigating related pharmacological interventions should take the potential impact of patients' blood lactate levels on the outcomes of these experiments into account.

### Neurotransmitter alteration

Neurotransmission mediated by acetylcholine (ACh) has long been regarded as contributing to numerous physiologic functions including memory, learning, and panic responses. The interaction between sepsis-induced cytokines and Ach is also believed to be associated with SAE. In particular, IL-1β inhibits ACh release and increases acetylcholinesterase activity and mRNA expression *in vivo*^55^. The reduction of cholinergic innervation could be detected by measuring the vesicular acetylcholine transporter 3 months after complete recovery from sepsis in an LPS-induced rat model^56^. A prospective study observed a significant increase in acetylcholinesterase (AChE) activity in septic patients experiencing cognitive dysfunction^57^. Together, these studies suggest ACh alteration secondary to sepsis plays a crucial role in SAE, especially in the long-term effects.

Glutamate, on the other hand, is the most prevalent excitatory neurotransmitter and is essential for maintaining neural function^58^. Recent studies focusing on changes in glutamate after sepsis and glutamate receptor-mediated neuro-excitotoxicity seem to be conflicting. Some studies have reported that glutamate accumulation in the synaptic space of hippocampal neurons after sepsis leads to neuronal excitotoxicity, with dexmedetomidine reducing glutamate accumulation and improving sepsis outcomes by activating α2 adrenergic receptors on astrocytes^59^. Conversely, other research has shown that enhancing glutamatergic excitatory projections in the medial prefrontal cortex-hippocampus pathway can improve cognitive function in SAE mice^60^. Further studies will be necessary to provide more accurate data for a clearer understanding of those results.

Other neurotransmitters, such as γ-aminobutyric acid (GABA) and 5-Hydroxytryptamine (5-HT), also exhibit alterations in SAE^24^, although recent studies on these changes are relatively limited. Conversely, the role of neurotransmitter mediated neuro-immune interactions in neuroinflammation and sepsis has attracted attention. A series of studies have indicated that GABA regulates the migration of monocytes and the inflammatory activation of macrophages^61^, ^62^. Exogenous administration of GABA can inhibit or maintain macrophage inflammatory response^61^. This bidirectional effect may depend on the maturation stage and the expression of GABA transporters (GAT) on macrophages. Specifically, GAT2 assists in maintaining the production of IL-1β. While the loss-of-function of GAT2 causes an increase in GAT4 expression, which further leads to a decrease in IL-1β expression^61^. Although the latest perspective suggests that microglia do not differentiate from bonemarrow hematopoietic stem cells, both originate from the yolk sac during the embryonic stage, thus sharing similar gene expression profiles^63^. And there are evidences indicating that enhancing GABA signaling can significantly inhibit the activation of microglia in a mouse model with intraperitoneal injection of LPS^64^, yet the specific underlying mechanism remains to be elucidated.

### Synapse loss and synaptic plasticity disorder

Dendritic reduction and synaptic plasticity disorder have been observed in both acute and chornic neuroinflammation conditions. Microglia are the primary excutors of synaptic pruning and phagocytosis of damaged synapses. As one of the most important DAMPs, high-mobility group box 1 (HMGB1) is mainly released by innate immune cells in sepsis. It has been proved that HMGB1 mediates synaptic loss in SAE mice^65^, and persistent elevated HMGB1 was observed at 4 weeks after CLP modeling^66^ suggesting HMGB1 might be associated with long-term cognitive dysfunction after sepsis. Yang Kun and his colleagues recently found that lactate was able to promote HMGB1 lactylation, leading to HMGB1 secretion and accumulation in sepsis mice^67^. Considering that a hyperlactic state is one of the common features of sepsis and septic shock and that the brain tends to uptake lactate as an emergency energy supply under an inflammation state, this finding may provide support for the argument of early lactate clearance. On the other hand, Ben Lv and his colleagues uncovered that heparan can interact with HMGB1 and suppress downstream caspase-11 signal, which is responsible for pyroptosis^68^, ^69^. They further found that sulfated non-anticoagulant heparin, a chemically modified heparin without anticoagulant activity, can also block caspase-11, which means this modified heparin may be able to clinically suppress neuroinflammation without considering the risk of hemorrhage.

The complement system is also involved in the excessive pruning of synapses by microglia. Several studies have shown that microglia clear C1q- and C3-labeled synapses^48^, ^65^, ^70^. In SAE, elevated HMGB1 leds to elevated C1q who further mediates excessive synaptic elimination by microglia^48^. The same process has also been observed in tau pathology^71^, suggesting that this may be a common mechanism of SAE and neurodegenerative diseases.

The formation and maintenance of synaptic plasticity rely on the dynamic changes of postsynaptic membrane receptors. Post-synaptic density protein 95 (PSD-95) is a scaffolding protein on the postsynaptic membrane of excitatory synapses and also an important protein that facilitates the trafficking of α-amino-3-hydroxy-5-methyl-4-isoxazole-propionicacid receptor (AMPAR) and N-methyl-D-aspartic acid receptor (NMDAR)^72^. A significant decrease in PSD-95 has been demonstrated in various animal models of SAE^73^-^75^. A recent study^76^ uncovered a novel pathway in regulating neuronal dysfunction by focusing on the interaction between hemoglobin subunit beta (Hbb) and PSD-95. Activated by sepsis-induced hypoxia, lncRNA nuclear enriched abundant transcript 1 (Neat1) binds with Hbb preventing it from ubiquitination and degradation. And the accumulated Hbb suppresses PSD-95 expression. Although Hbb has recently been found to be expressed in neurons and glial cells, the peripheral erythrocyte seems to be the main source of circulating Hbb in sepsis. Therefore, it is logical to assume that circulating Hbb may be responsible for PSD-95 reduction through disrupted BBB.

Another essential factor that contributes to synaptic plasticity is the neuronal growth factor brain-derived neurotrophic factor (BDNF). Experimental BDNF sequestration has been demonstrated to contribute to cognitive impairment by blocking the hippocampal long-term potentiation (LTP)^77^. BDNF/ BDNF receptor tyrosine receptor kinase B (TrκB) signaling has long been proven to be the LTP development pathway^78^. The activation and protection methods for the BDNF/TrκB signaling pathway are hot topics in the study of SAE. Heparan sulfate is a linear polysaccharide with the capacity to interact with soluble proteins and is abundant on the cell surface and extracellular matrix. A recent study demonstrated that circulating heparan sulfate might inhibit BDNF/TrκB signaling by specifically binding to BDNF, as the administration of TrκB agonists effectively ameliorates LPT in mice^79^. Another study found that heparan sulfate selectively targeted and penetrated the hippocampal BBB following sepsis while sparing the cortex and other nonneuronal tissues^80^.

Taken together, recent studies have highlighted that peripheral tissues/systems, such as blood and vascular endothelium matrix, exhibit pathological changes in sepsis that affect the nervous system and ultimately induce SAE.

## Diagnostic methods for SAE

### Sepsis diagnosis and SAE suspicion

As is defined, SAE is a syndrome secondary to sepsis in the absence of direct CNS infection, structural abnormality, or other types of encephalopathy. The first step in the diagnosis of SAE is to seek evidence of sepsis or sepsis suspicion and then to exclude other types of encephalopathy. The criteria for “Sepsis-3” criteria stipulate that the diagnostic criteria for sepsis are a Sequential Organ Failure Assessment (SOFA) score of 2 or greater caused by infection^1^.

However, as a common symptom of SAE, delirium may occur before the definite diagnosis of sepsis^3^. Patients with a quick SOFA score of 2 or greater with psychiatric symptoms also need to be alert to SAE. Encephalopathy, on the other hand, can be caused by a variety of inducements and have similar clinical manifestations. A prospective cohort study^81^ involving 1040 ICU patients shows that the most common inducements of delirium were anesthesia, sepsis, hypoxia, and metabolic disorders (hepatic encephalopathy, renal encephalopathy, hypoglycemia, dehydration, etc.), and two or more phenotypes of encephalopathy often co-occurred especially at the acute phase of sepsis. Therefore, it is argued that the diagnosis of SAE should exclude confounding factors such as liver and kidney dysfunction, glucose and lipid metabolism disorders, and drug-induced delirium. However, those abnormalities also reflect the severity of sepsis, which is associated with the increasing risk of SAE. And it is challenging to rule out all the confounding factors in the course of sepsis. In this case, we believe that the diagnosis treatment may be helpful to identify SAE. For example, it should be more suspicious of SAE if delirium symptoms or other neurological indicators do not improve or even worsen after glycemic control or shock resuscitation. This approach is similar to fluid challenge testing to assess fluid responsiveness in patients with shock, but it requires dynamic quantification of delirium and neurologic function.

### Scoring systems and prediction models for SAE and delirium

At present, there is still a lack of a delirium scoring system specifically for sepsis patients, and most studies use the delirium scoring system for critically ill patients to score sepsis patients. The Confusion Assessment Method (CAM) has been validated for use in the diagnosis of delirium for patients outside the ICU and showed a sensitivity of 94-100% and specificity of 90-95%^82^. However, CAM is not suitable for patients who cannot speak. For those patients who are treated in the ICU or during the perioperative period with sedative drug usage, there developed the CAM-ICU system. The ingredient of the Richmond Agitation Sedation Scale (RASS) makes CAM-ICU more specific for delirium with a specificity of 98-100%, but less sensitive (41-53%) and harder to get mastered^83^. Nevertheless, CAM-ICU may not be the optimal screening tool for early delirium screening in older acute patients, since the delirium prevalence using the CAM-ICU is much lower than the expected prevalence in a prospective cohort study^84^. Some other scoring systems were less validated in sepsis patients, and we present the details of those systems in Table 1.

To recognize delirium earlier, prediction models have been developed clinically. The PRE-DELIRIC (PREdiction of DELIRIum in ICu patients) model is the first validated prediction model for intensive care patients developed in 2012 and shows a high predictive value in delirium prediction for patients within 24 hours after ICU admission^85^. Based on this model, the Early-PRE-DELIRIC (E-PRE-DELIRIC) was developed and validated by a multinational study in 2015 for predicting delirium at the time of ICU admission^86^. However, a recent large sample retrospective validation in a UK general ICU indicates that the utility of E-PRE-DELIRIC for guiding clinical decision-making is limited since its positive predictive value is only slightly higher than delirium incidence^87^.

With the development of artificial intelligence and big data, risk prediction models based on machine learning have also been used for early identification of delirium^88^-^90^. By reviewing 4 quantitatively analyzed studies on machine learning-based models, a meta-analysis revealed the overall pooled area under the receiver operating characteristic curve for predicting delirium was 0.89, sensitivity 85%, and specificity 80%^91^. Compared with the traditional risk prediction formula calculated by logistic regression analysis, machine learning is a set of autonomous learning and prediction systems based on computer algorithms, and the model formula can be automatically updated in the application stage to maintain high sensitivity and specificity. Meanwhile, factors such as changes in clinical guidelines and different population characteristics make the prediction models calculated by logistic regression have a higher risk of bias, which makes it difficult to establish a set of mathematical models with wide applicability. Most importantly, it is difficult to diagnose SAE by scoring or predictive models alone because they can only determine the presence or risk of delirium at one single timepoint in the course of disease. Through AI recognition and dynamic capture of clinical data (such as analgesic drugs, blood pressure, blood sugar and other indicators), the machine based-learning model can dynamically assess delirium while reducing the impact of confounding factors. If the risk of delirium continues to increase during sepsis or neural damage does not recover with remission of sepsis, the more specific the diagnosis of SAE will be. Overall, we believe that machine learning-based models have a strong application prospect in assisting clinical decision-making. However, the first and most resistant step is to establish a widely covered, standardized electronic medical records database to allow the algorithm to automatically learn and update the model. Of note, due to the lack of standardized databases, none of these existing machine learning-based prediction models for SAE have been externally validated^91^.

### Biomarkers for SAE

A golden biomarker or biomarkers combination should be accurate and reproducible with both high specificity and sensitivity. As presented in the mechanisms section, SAE may be associated with a variety of intercellular or intracellular alterations. Biomarkers related to those alterations have been studied in clinical trials. Calcium-binding protein β (S100β) is a neuron-specific serum biomarker reflecting BBB disruption, neuroglia injury, and activation. The increase of S100β on day 3 after ICU admission was recently observed to be independently correlated with SAE in a prospective cohort study^92^. Furthermore, by setting the cut-off level of 0.144 μg/L (area under the curve (AUC) was 0.819), S100β on day 3 presented 84.44% specificity and 69.49% sensitivity in SAE diagnosis. Another classic neuron-specific biomarker for SAE is neuron-specific enolase (NSE). Although the diagnostic and prognostic value of NSE is not as good as that of S100β in SAE^93^, the combination of NSE and S100β showed good diagnostic specificity and sensitivity in the diagnosis and outcome prediction of other neurological injury diseases^94^, ^95^. It is reasonable to hypothesize that a combination of a range of biomarkers related to nerve injury could further improve the efficiency of SAE diagnosis.

However, considering that patients with sepsis may be accompanied by hypotension or even shock, a differential diagnosis between SAE and ischemic brain injury is warranted. Previously, Johannes Ehler et al performed a longitudinal prospective translational study, proving that ischemic lesions and neuroaxonal injury could be consistently found in both septic rats and human brains^96^. This promotes neurofilament proteins as upcoming biomarker candidates in delirium and SAE. The authors further conducted another clinical trial, suggesting that plasma neurofilament light chain (NFL) was significantly higher in patients with SAE and correlated with the severity of SAE^97^. More recently, plasma NFL showed remarkable prognostic value among critically ill patients^98^.

### Electroencephalogram for SAE

The change in electroencephalogram (EEG) is another indicator of SAE diagnosis and severity. It's reported that 50% of sepsis cases have abnormalities on EEG, and this change is reversible when sepsis is effectively treated^3^. A prospective cohort study including 102 ICU patients with sepsis or septic shock suggested that delirium was associated with a preponderance of low-frequency continuous EEG (cEEG) activity and the absence of high-frequency cEEG activity^99^. Mild encephalopathy is associated with the slowing of brain activity in the theta range and severe encephalopathy is usually associated with excessive delta waves or a burst-suppression pattern of activity. Three-phase waves, commonly seen in hepatic or renal encephalopathy, are also seen in about 20% of patients with SAE^3^, ^100^, and indicate a higher 1-year mortality rate^101^. Although EEG monitoring is the most sensitive tool for evaluating brain function, the pleomorphism of EEG makes it play an auxiliary role in the diagnosis of SAE, especially when sedative drugs are used. In a prospective study of pediatric SAE, the application of distinct EEG diagnostic criteria led to a substantial variation in the detection rate of SAE (26.9% with Criterion A [strict criteria requiring focal slowing, epileptiform discharges, or periodic patterns] versus 96.2% with Criterion B [lenient criteria based on background theta/delta waves as encephalopathic markers])^102^. Above all, these studies indicated EEG may provide great help for SAE diagnosis, but need further exploration.

### Image tools for SAE

Current imaging tests and studies in SAE focus on CT and MRI. For clinical practice purposes, CT is relatively more commonly used since part of SAE patients require maintenance of sedation and continuous intravenous vasoactive drugs, which are inoperable for MRI. Imaging findings of SAE include brain atrophy, white matter hyperintensities, edema, cortical or subcortical hemorrhage, or complete normality due to the inconsistencies in clinical manifestations of SAE and interference factors such as pathogens and treatments^103^, ^104^. Other imaging tools, such as functional MRI, magnetic resonance spectroscopy (MRS), and PET, are rarely used in the clinical diagnosis of SAE but play a great role in the studies of the pathogenesis of SAE. Default mode network which mainly includes the precuneus, posterior cingulate cortex, inferior parietal, medial prefrontal cortex, and hippocampus, has been proven to present altered connectivity in LPS-induced SAE rats by resting-state functional MRI^105^. In line with other neuropsychiatric disorder diseases such as post-traumatic stress disorder and chronic kidney failure-related dementia^106^, ^107^, resting-state functional MRI may be helpful for the localization of SAE-related brain functional regions. Based on magnetic resonance technology, the new generation imaging system integrated spectrometry analysis derived MRS, which can noninvasively detect the changes of metabolites in the brain of SAE animal models^108^. Apart from that, [^11^C]PBR28, a radioligand with high affinity for the 18kD translocator protein which is associated with microglial activation, has been validated in measuring neuroinflammation by PET^109^, ^110^. Most recently, Jie Xiang and her colleagues developed a novel PET tracer for a-synuclein^111^, which has been proven to be elevated in sepsis brain^112^. This or some upcoming novel tracer may be of high value in the diagnosis of a group of neurodegenerative diseases including SAE.

## Treatment and management for SAE

Despite the ongoing insights into SAE, there are still no specific, evidence-based therapeutic options for the treatment of SAE in patients. Considering that SAE occurs secondary to sepsis with the absence of direct CNS infection, the treatment remains focused on preventing the occurrence of SAE by treating sepsis and suppressing systematic inflammatory response syndrome (SIRS). Statistics from 1979 to 2000 in the United States show that bacterial infections account for 90% of all sepsis cases, with 52% of all cases caused by Gram-positive bacteria and 38% by Gram-negative bacteria, and polymicrobial and fungal infections accounting, respectively, for 4.7% and 4.6% of all cases^113^. In addition, the virus is also capable of inducing sepsis by definition. COVID-19 caused approximately 5% of patients to suffer critical manifestations defined as respiratory failure, septic shock, and multiple organ dysfunction^114^. Therefore, broad-spectrum and specific-spectrum antimicrobial agents are necessary. Other treatment methods for sepsis and septic shocks such as fluid resuscitation, vasoactive drug administration, glycemia control, and nutritional support are also recommended^115^.

It is worth mentioning that the usage of corticosteroids in patients with sepsis remains controversial^116^. Guidelines from the European Society of Intensive Care Medicine and the Society of Critical Care Medicine recommend using corticosteroids in adult sepsis patients with septic shock and an ongoing requirement for vasopressor therapy^6^. A well-designed study used a multicenter, randomized, double-blind, positive drug-parallel control design and included 34 ICUs in university hospitals and community hospitals in Germany, with a total of 190 cases collected in each group. It finds no reduction in the risk of septic shock within 14 days among adults using hydrocortisone for severe sepsis^117^. Interestingly, delirium was found to develop less frequently in patients treated with low-dose hydrocortisone in this study. Relatively, high-dose corticosteroids have long been found to be associated with altering the function and morphology of the hippocampus, and further causing cognitive impairment. In general, the strategy of corticosteroid use specifically in SAE treatment needs further study.

Sedative medication is a common confounder in SAE diagnosis and treatment. Clinical practice guidelines about sedation recommend <1> using short-acting sedative medications such as propofol or dexmedetomidine; <2> monitoring depth of sedation using a validated scale such as RASS and Sedation Analgesia Scale; <3> maintaining light levels of sedation; <4> stopping continuous sedative medications at least once daily to allow patients to awaken and be reoriented; and <5> monitoring for delirium regularly using a validated scale such as CAM-ICU^115^. Dexmedetomidine is a widely used α2 adrenoceptor agonist to provide sedation. Further clinical trials revealed its neuroprotective effects and beneficial effect on neurocognitive function with lower risk and shorter duration of mechanical ventilation and delirium in critically ill (not specifically in sepsis or SAE) patients^118^, ^119^. A recent study proves that the systemic administration of dexmedetomidine can attenuate SAE and sepsis-associated inflammation through α2A adrenoceptors in astrocytes in CLP-induced mouse model^59^. On the other hand, propofol, as another clinically widely used sedative, has the characteristics of rapid induction of anesthesia and a short half-life, which is often used in clinical trials to compare with dexmedetomidine. A multicenter, double-blind, randomized controlled clinical study with a strict quality control design enrolling 422 patients, tells no significant difference between dexmedetomidine and propofol on days alive without delirium or coma, ventilator-free days, death at 90 days, and cognitive status score at 6 months in mechanically ventilated adults with sepsis^120^. Besides, a recently published meta-analysis of randomized controlled trials on mechanical ventilation patients in ICU draws a similar conclusion on the sepsis subset but emphasizes the increased risk of bradycardia induced by dexmedetomidine^121^. Some of the clinical trials in sepsis or sepsis shock patients about sedative drugs in the last 10 years are present in Table 2.

On the other hand, statistical data from 1996 to 2008 in the United States shows approximately 74.7% of over 3-year sepsis survivors suffered functional disability and 16.7% suffered moderate to severe cognitive impairment^122^. The relatively new data come from Germany^123^, a population-based study involving 116,507 sepsis survivors between 2013 and 2017 demonstrated significant post-sepsis morbidity, with 74.3% developing new medical, psychological, or cognitive disorders within one year of discharge. Notably, 18.5% of survivors were newly diagnosed with cognitive impairment, rising to 28.5% in survivors older than 80 years. Functional outcomes were similarly concerning, as 31.5% of survivors without pre-existing dependency required new nursing care services. Economically, the cumulative three-year healthcare costs averaged €29,088 (about $32,000 USD) per patient, underscoring the substantial societal burden. These findings highlight the enduring multi-domain impact of sepsis, particularly cognitive decline (which can be diagnosed with SAE) in elderly populations, and emphasize the need for integrated post-discharge rehabilitation strategies targeting functional recovery and long-term health maintenance. A cohort study involving 15,535 post-sepsis patients presents a significantly lower risk of 10-year mortality by receiving rehabilitation (including facilitating muscle strengthening and movement, activities of daily living, cardiovascular capacity, functional ability, and occupational and communication therapy) within 90 days after discharge^124^. The guideline suggests^6^, but not recommend rehabilitation programs for sepsis survivors. Meanwhile, it proposes future research to determine an optimal approach to functional rehabilitation (timing, dosing, intensity, and duration) and patient selection.

## Current issues in SAE research

Despite extensive clinical research focusing on delirium and neurological outcomes in sepsis patients, as previously discussed, the concept of SAE remains underutilized in high-quality studies. The primary barrier is the lack of standardized diagnostic criteria for SAE, which hinders rigorous case inclusion in clinical trials. While prospective studies may adopt current exclusion-based diagnostic criteria for SAE, their generalizability is compromised because patients with comorbid encephalopathies (frequently overlapping with SAE) are systematically excluded. Retrospective studies relying on electronic health records or databases face even greater challenges due to pervasive data incompleteness and misclassification. Moreover, the ethical and legal issues involved in medical data sharing also deserve attention^125^.

Ethical complexities further complicate SAE research. First, SAE patients often exhibit impaired capacity to provide informed consent, necessitating proxy consent from legal guardians^126^. Second, vulnerable populations such as pediatric and maternal cohorts, who account for over 14% of annual sepsis cases (notably children under five)^127^, are frequently excluded from trials to mitigate risks. However, this exclusion undermines the external validity of findings, given the high sepsis burden in these groups.

The principle of non-maleficence is one of the basic principles of clinical ethics, which also applies to the research and treatment of SAE. Despite the long-term risk of SAE for sepsis survivors, the priority for treatment of sepsis remains to be saving lives. Theoretically, SAE could benefit from anti-inflammatory therapy at any stage of sepsis since SAE patients do not have a direct CNS infection and the roles in assisting pathogen clearance of inflammatory cytokines are not required.

However, a number of previous studies have shown that antagonists/antibodies of inflammatory cytokines such as TNF-α and IL-1β^128^ and antagonists TLR4-MD2 complex^129^ fail to benefit patients with sepsis, and even aggravate immunesuppression in some patients with severe sepsis. Due to the presence of the BBB, the immune environment of the CNS is relatively independent, while peripheral immune disorders caused by sepsis can lead to the continuous or simultaneous occurrence of SIRS and compensatory anti-inflammatory response syndrome (CARS)^130^, which will lead to potential discordance between neuroinflammatory processes and peripheral immune status. Therefore, SAE clinical studies should also consider the impact of intervention protocols on sepsis especially when attempting to treat SAE with systemic interventions. And any intervention that may amplify the clinical risk of enrolled sepsis patients should be withdrawn immediately.

Animal studies encounter parallel limitations. A critical issue is the absence of standardized SAE models. Most studies define SAE in rodents as sepsis survivors with neurological deficits, typically induced by CLP or LPS injection. However, sepsis severity varies widely across protocols, with mortality rates ranging from 50% to 80% during model induction^131^. Moreover, surviving animals do not uniformly develop to SAE, raising concerns about reproducibility and ethical compliance with the 3R principles (Replacement, Reduction, Refinement). Notably, certain ethics committees have prohibited CLP models due to excessive animal suffering^132^. Heterogeneity in experimental designs also contributes to conflicting results. For instance, while some studies report hippocampal neuronal death in SAE mice, others fail to replicate this finding. In response, the SSC work groups have prioritized the development of standardized sepsis models to establish reliable SAE frameworks^132^. Addressing these methodological and ethical challenges is essential to advance translational research and ensure clinically relevant insights into SAE pathophysiology and therapeutic interventions.

## Conclusion and Prospects

**Figure 3:**
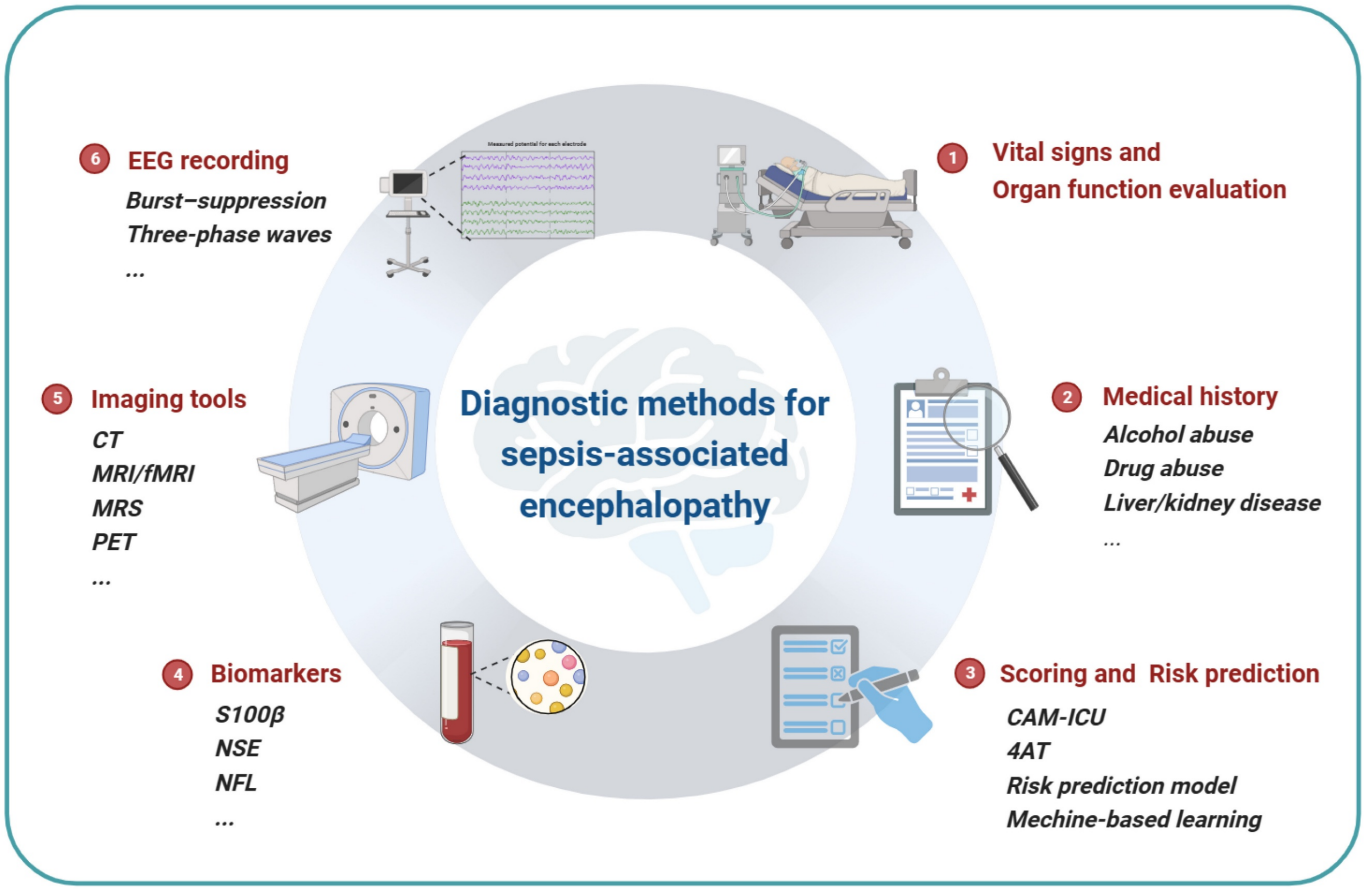
** Diagnostic methods for SAE.** The summary of current diagnostic methods of sepsis-associated encephalopathy. Vital signs and organ function evaluation is the foundational requirement of sepsis diagnosis. The acknowledgment of medical history is necessary for the risk and outcome prediction, which can be qualified by a scoring system or risk prediction model. Neuron damage sensitive biomarkers such as S100β, NSE and NFL are serum indicators of SAE. Imaging tools such as CT and MRI are widely used to evaluate neural damage in clinical practice, and MRS and PET can be used to detect metabolite changes in the brain. Continuous EEG recording can reflect the functional changes of the brain.

It is clear that sepsis-associated encephalopathy is a severe complication of sepsis and is highly related to the mortality and the neurofunction outcome of the patients. However, since the mechanisms of SAE are complex with multiple pathways involved and the difference between animal models and clinical patients, researches based on animal models are helpful to clarify the mechanisms of SAE, but only a few are able to guide clinical diagnosis or treatment. On the other hand, a valid and unified diagnosis protocol for SAE is urgently needed and essential for clinical trials to make the results comparable. The novel imaging tools, biomarkers and electroencephalograms are potential indicators. And the recently validated scoring system or prediction models based on artificial intelligence and big data may guide clinical practice by early identification or differentiation of SAE phenotypes (Fig 3). Finally, there is no solid evidence for specific treatments for SAE yet, while for the long-term sequela, functional rehabilitation is essential.

## Figures and Tables

**Table 1:** Advantages and limitations of scoring systems and models in identifying delirum for sepsis patients

Tool/Model	Included clinical indicators/scoring criteria	Target Population	Advantages	Limitations	Performance Metrics	References
**CAM-ICU**	Consciousness, attention, thinking, alertness (based on 4-item assessment)	ICU patients (especially mechanically ventilated)	Simple to use, suitable for non-verbal patients; high specificity	Requires trained staff; may miss hypoactive delirium	Sensitivity 0.86, specificity 0.77.	Ely et al.^133^
**ICDSC**	8 items: Altered level of consciousness, inattention, disorientation, hallucinations/delusions, psychomotor agitation/retardation, inappropriate speech/mood, sleep-wake cycle disturbance, symptom fluctuation. Score ≥4 indicates delirium.	ICU patients (excluding coma or pre-existing delirium)	High sensitivity (99%); feasible for clinicians/nurses in busy ICU settings; applicable to nonverbal patients.	Lower specificity (64%); high false positives in patients with psychiatric/neurological conditions.	Sensitivity 0.99, Specificity 0.64, AUROC=0.9017 (ROC analysis)	Bergeron et al.^134^
**PRE-DELIRIC**	10 risk factors: Age, APACHE-II score, admission group (medical/surgical/trauma/neurology), coma status (drug-induced/miscellaneous/combination), infection, metabolic acidosis, morphine dose categories, sedative use, urea concentration, urgent admission.	ICU patients within 24 hours of admission	High discriminative power (AUROC=0.85 pooled); validated in multinational cohorts; dynamic risk stratification.	Requires complete clinical data; static model (does not update with ICU stay changes); external validation variability.	AUROC=0.87 (development), Calibration slope=0.93 (pooled).	Boogaard et al.^85^
**E-PRE-DELIRIC**	5 risk factors: Age, APACHE-II score, admission category, infection, urea concentration.	ICU patients at admission	Simplified for rapid early assessment; requires minimal data input.	Poor calibration (underestimates risk, β=0.58); low PPV (~43.7%); limited generalizability in surgical cohorts.	AUROC=0.628-0.648 (UK validation)Calibration slope β=0.58 (95% CI 0.46-0.71).	Wassenaar et al.^86^ Cowan et al.^87^
**Machine Learning Models**	Various indicators such as: Age, APACHE II/SOFA scores, inflammatory markers (CRP, IL-6), sedative use, mechanical ventilation duration	ICU patients with high data completeness	Handles nonlinear relationships and high-dimensional data; dynamic prediction	Relies on data quality; black-box models require explainability tools	Random forest/XGBoost AUROC up to 0.85-0.92 (varies across studies)	Xie et al.^91^

ICDSC: Intensive Care Delirium Screening Checklist; PPV: Positive Predictive Value

**Table 2:** Clinical trials of sedation in sepsis and sepsis shock patients

Author	Type of research	Sedation	Sedation protocol	Objects	Conclusion
Penna, et al.^135^	Prospective	Propofol Midazolam	Propofol 5μg/kg/hr, titrated 1-2mg/hr in day 1. Followed by midazolam 0.05 mg/kg(loading), 1-2 mg/hr(maintenance), titrated 1-2mg/hr.	Patients (n=16) with septic shock	Sublingual microcirculatory perfusion improved when the infusion was changed from propofol to midazolam in patients with septic shock. This observation could not be explained by changes in systemic hemodynamics.
Hughes, et al.^120^	RCT	DEX Propofol	DEX 0.2 to 1.5μg/kg/hr, propofol 5-50μg/kg/min	Mechanically ventilated adults (n=422) with sepsis	Among mechanically ventilated adults with sepsis who were being treated with recommended light-sedation approaches, outcomes in patients who received dexmedetomidine did not differ from outcomes in those who received propofol.
Ohta, et al.^136^	RCT	DEX	RASS score of 0 during the day and -2 during the night	ICU patients (n=201) with sepsis requiring mechanical ventilation	Sedation using dexmedetomidine reduced inflammation in patients with sepsis requiring mechanical ventilation.
Miyamoto, et al.^137^	RCT	DEX	RASS score of 0 during the day and -2 during the night	Patients (n=201) with sepsis shock	Among mechanically ventilated patients with septic shock, sedation with dexmedetomidine resulted in increased lactate clearance compared with sedation without dexmedetomidine.
Kawazoe, et al.^118^	RCT	DEX	RASS score of 0 during the day and -2 during the night	Patients (n=201) with sepsis undergoing ventilation	Among patients requiring mechanical ventilation, the use of dexmedetomidine compared with no dexmedetomidine did not result in statistically significant improvement in mortality or ventilator-free days.
Morelli, et al.^138^	Crossover Trial	DEX Propofol	RASS score of -3 to -4	Septic shock patients (n=38) requiring norepinephrine	For a comparable level of sedation, switching from propofol to dexmedetomidine resulted in a reduction of catecholamine requirements in septic shock patients.

RCT: Randomized clinical trial; RASS: Richmond Agitation Sedation Scale; DEX: Dexmedetomidine

## References

[1] Singer M, Deutschman CS, Seymour CW (2016). The Third International Consensus Definitions for Sepsis and Septic Shock (Sepsis-3). JAMA.

[2] Rudd KE, Johnson SC, Agesa KM (2020). Global, regional, and national sepsis incidence and mortality, 1990-2017: analysis for the Global Burden of Disease Study. Lancet.

[3] Gofton TE, Young GB (2012). Sepsis-associated encephalopathy. Nat Rev Neurol.

[4] Sonneville R, de Montmollin E, Poujade J (2017). Potentially modifiable factors contributing to sepsis-associated encephalopathy. Intensive Care Med.

[5] Iwashyna TJ, Ely EW, Smith DM, Langa KM (2010). Long-term cognitive impairment and functional disability among survivors of severe sepsis. JAMA.

[6] Evans L, Rhodes A, Alhazzani W (2021). Surviving sepsis campaign: international guidelines for management of sepsis and septic shock 2021. Intensive Care Med.

[7] Erikson K, Tuominen H, Vakkala M (2020). Brain tight junction protein expression in sepsis in an autopsy series. Crit Care.

[8] Peng X, Luo Z, He S, Zhang L, Li Y (2021). Blood-Brain Barrier Disruption by Lipopolysaccharide and Sepsis-Associated Encephalopathy. Front Cell Infect Microbiol.

[9] Wei C, Jiang W, Wang R (2024). Brain endothelial GSDMD activation mediates inflammatory BBB breakdown. Nature.

[10] Cuadrado A (2022). Brain-Protective Mechanisms of Transcription Factor NRF2: Toward a Common Strategy for Neurodegenerative Diseases. Annu Rev Pharmacol Toxicol.

[11] Chen S, Tang C, Ding H (2020). Maf1 Ameliorates Sepsis-Associated Encephalopathy by Suppressing the NF-*k*B/NLRP3 Inflammasome Signaling Pathway. Front Immunol.

[12] Liu J, Jin Y, Ye Y (2021). The Neuroprotective Effect of Short Chain Fatty Acids Against Sepsis-Associated Encephalopathy in Mice. Front Immunol.

[13] You L, Jiang H (2021). Cabergoline possesses a beneficial effect on blood-brain barrier (BBB) integrity against lipopolysaccharide (LPS). Bioengineered.

[14] Pu Y, Zhao L, Xi Y, Xia Y, Qian Y (2022). The protective effects of Mirtazapine against lipopolysaccharide (LPS)-induced brain vascular hyperpermeability. Bioengineered.

[15] Cui W, Chen J, Yu F, Liu W, He M (2021). GYY4137 protected the integrity of the blood-brain barrier via activation of the Nrf2/ARE pathway in mice with sepsis[J]. FASEB J.

[16] Dal-Pizzol F, Rojas HA, dos Santos EM (2013). Matrix metalloproteinase-2 and metalloproteinase-9 activities are associated with blood-brain barrier dysfunction in an animal model of severe sepsis. Mol Neurobiol.

[17] Zhang N, Zhao W, Hu ZJ (2021). Protective effects and mechanisms of high-dose vitamin C on sepsis-associated cognitive impairment in rats. Sci Rep.

[18] Lamontagne F, Masse MH, Menard J (2022). Intravenous Vitamin C in Adults with Sepsis in the Intensive Care Unit. N Engl J Med.

[19] Moraes CA, Zaverucha-do-Valle C, Fleurance R, Sharshar T, Bozza FA, d'Avila JC (2021). Neuroinflammation in Sepsis: Molecular Pathways of Microglia Activation. Pharmaceuticals (Basel).

[20] Erickson MA, Banks WA (2018). Neuroimmune Axes of the Blood-Brain Barriers and Blood-Brain Interfaces: Bases for Physiological Regulation, Disease States, and Pharmacological Interventions. Pharmacol Rev.

[21] Zhu Y, Zhang Y, He S (2024). Integrating single-nucleus RNA sequencing and spatial transcriptomics to elucidate a specialized subpopulation of astrocytes, microglia and vascular cells in brains of mouse model of lipopolysaccharide-induced sepsis-associated encephalopathy. J Neuroinflammation.

[22] Michels M, Vieira AS, Vuolo F (2015). The role of microglia activation in the development of sepsis-induced long-term cognitive impairment. Brain Behav Immun.

[23] Qu H, Wu J, Pan Y (2024). Biomimetic Nanomodulator Regulates Oxidative and Inflammatory Stresses to Treat Sepsis-Associated Encephalopathy. ACS Nano.

[24] Li Y, Ji M, Yang J (2022). Current Understanding of Long-Term Cognitive Impairment After Sepsis. Front Immunol.

[25] Lorne E, Dupont H, Abraham E (2010). Toll-like receptors 2 and 4: initiators of non-septic inflammation in critical care medicine?. Intensive Care Med.

[26] Manabe T, Heneka MT (2022). Cerebral dysfunctions caused by sepsis during ageing. Nat Rev Immunol.

[27] Xi S, Wang Y, Wu C, Peng W, Zhu Y, Hu W (2022). Intestinal Epithelial Cell Exosome Launches IL-1β-Mediated Neuron Injury in Sepsis-Associated Encephalopathy. Front Cell Infect Microbiol.

[28] Hueston CM, O'Leary JD, Hoban AE (2018). Chronic interleukin-1β in the dorsal hippocampus impairs behavioural pattern separation. Brain Behav Immun.

[29] Wright SS, Kumari P, Fraile-Ágreda V (2025). Transplantation of gasdermin pores by extracellular vesicles propagates pyroptosis to bystander cells. Cell.

[30] Endo F, Kasai A, Soto JS (2022). Molecular basis of astrocyte diversity and morphology across the CNS in health and disease. Science.

[31] Zhang L, Jia Z, Wu Q (2023). Alleviating symptoms of neurodegenerative disorders by astrocyte-specific overexpression of TMEM164 in mice. Nat Metab.

[32] Kwon HS, Koh SH (2020). Neuroinflammation in neurodegenerative disorders: the roles of microglia and astrocytes. Transl Neurodegener.

[33] Guo Q, Gobbo D, Zhao N (2024). Adenosine triggers early astrocyte reactivity that provokes microglial responses and drives the pathogenesis of sepsis-associated encephalopathy in mice. Nat Commun.

[34] Simats A, Zhang S, Messerer D (2024). Innate immune memory after brain injury drives inflammatory cardiac dysfunction. Cell.

[35] Wendeln AC, Degenhardt K, Kaurani L (2018). Innate immune memory in the brain shapes neurological disease hallmarks. Nature.

[36] Barichello T, Sayana P, Giridharan VV (2019). Long-Term Cognitive Outcomes After Sepsis: a Translational Systematic Review. Mol Neurobiol.

[37] Boede M, Gensichen JS, Jackson JC (2021). Trajectories of depression in sepsis survivors: an observational cohort study. Crit Care.

[38] Manfredini A, Constantino L, Pinto MC (2019). Mitochondrial dysfunction is associated with long-term cognitive impairment in an animal sepsis model. Clin Sci (Lond).

[39] Sharshar T, Gray F, Lorin de la Grandmaison G (2003). Apoptosis of neurons in cardiovascular autonomic centres triggered by inducible nitric oxide synthase after death from septic shock. Lancet.

[40] Ulfig A, Jakob U (2024). Cellular oxidants and the proteostasis network: balance between activation and destruction. Trends Biochem Sci.

[41] Yauger YJ, Bermudez S, Moritz KE, Glaser E, Stoica B, Byrnes KR (2019). Iron accentuated reactive oxygen species release by NADPH oxidase in activated microglia contributes to oxidative stress in vitro. J Neuroinflammation.

[42] Gao Y, Tu D, Yang R, Chu CH, Hong JS, Gao HM (2020). Through Reducing ROS Production, IL-10 Suppresses Caspase-1-Dependent IL-1β Maturation, thereby Preventing Chronic Neuroinflammation and Neurodegeneration. Int J Mol Sci.

[43] Dumbuya JS, Li S, Liang L, Chen Y, Du J, Zeng Q (2022). Effects of hydrogen-rich saline in neuroinflammation and mitochondrial dysfunction in rat model of sepsis-associated encephalopathy. J Transl Med.

[44] Cui Y, Meng S, Zhang N (2024). High-concentration hydrogen inhalation mitigates sepsis-associated encephalopathy in mice by improving mitochondrial dynamics. CNS Neurosci Ther.

[45] Devine MJ, Kittler JT (2018). Mitochondria at the neuronal presynapse in health and disease. Nat Rev Neurosci.

[46] Yin L, Zhang J, Ma H (2023). Selective activation of cholinergic neurotransmission from the medial septal nucleus to hippocampal pyramidal neurones improves sepsis-induced cognitive deficits in mice. Br J Anaesth.

[47] Grünewald B, Wickel J, Hahn N (2024). Targeted rescue of synaptic plasticity improves cognitive decline in sepsis-associated encephalopathy. Mol Ther.

[48] Chung HY, Wickel J, Hahn N (2023). Microglia mediate neurocognitive deficits by eliminating C1q-tagged synapses in sepsis-associated encephalopathy. Sci Adv.

[49] McCubbin S, Meade A, Harrison DA, Cooper RL (2024). Acute lipopolysaccharide (LPS)-induced cell membrane hyperpolarization is independent of voltage gated and calcium activated potassium channels. Comp Biochem Physiol C Toxicol Pharmacol.

[50] Lin MM, Liu N, Qin ZH, Wang Y (2022). Mitochondrial-derived damage-associated molecular patterns amplify neuroinflammation in neurodegenerative diseases. Acta Pharmacol Sin.

[51] Yang Y, Ke J, Cao Y, Gao Y, Lin C (2024). Melatonin regulates microglial M1/M2 polarization via AMPKα2-mediated mitophagy in attenuating sepsis-associated encephalopathy. Biomed Pharmacother.

[52] Ding H, Li Y, Chen S (2022). Fisetin ameliorates cognitive impairment by activating mitophagy and suppressing neuroinflammation in rats with sepsis-associated encephalopathy. CNS Neurosci Ther.

[53] Hayakawa K, Esposito E, Wang X (2016). Transfer of mitochondria from astrocytes to neurons after stroke. Nature.

[54] Zhou J, Zhang L, Peng J (2024). Astrocytic LRP1 enables mitochondria transfer to neurons and mitigates brain ischemic stroke by suppressing ARF1 lactylation. Cell Metab.

[55] Zhang QH, Sheng ZY, Yao YM (2014). Septic encephalopathy: when cytokines interact with acetylcholine in the brain. Mil Med Res.

[56] Semmler A, Frisch C, Debeir T (2007). Long-term cognitive impairment, neuronal loss and reduced cortical cholinergic innervation after recovery from sepsis in a rodent model. Exp Neurol.

[57] Zujalovic B, Mayer B, Hafner S, Balling F, Barth E (2020). AChE-activity in critically ill patients with suspected septic encephalopathy: a prospective, single-centre study. BMC Anesthesiol.

[58] Verma M, Lizama BN, Chu CT (2022). Excitotoxicity, calcium and mitochondria: a triad in synaptic neurodegeneration. Transl Neurodegener.

[59] Mei B, Li J, Zuo Z (2021). Dexmedetomidine attenuates sepsis-associated inflammation and encephalopathy via central α2A adrenoceptor. Brain Behav Immun.

[60] Ge C, Chen W, Zhang L, Ai Y, Zou Y, Peng Q (2023). Chemogenetic activation of the HPC-mPFC pathway improves cognitive dysfunction in lipopolysaccharide -induced brain injury. Theranostics.

[61] Xia Y, He F, Wu X (2021). GABA transporter sustains IL-1β production in macrophages. Sci Adv.

[62] Fu J, Han Z, Wu Z (2022). GABA regulates IL-1β production in macrophages. Cell Rep.

[63] Huang Y, Xu Z, Xiong S (2018). Repopulated microglia are solely derived from the proliferation of residual microglia after acute depletion. Nat Neurosci.

[64] Jiang J, Tang B, Wang L (2022). Systemic LPS-induced microglial activation results in increased GABAergic tone: A mechanism of protection against neuroinflammation in the medial prefrontal cortex in mice. Brain Behav Immun.

[65] Yin XY, Tang XH, Wang SX (2023). HMGB1 mediates synaptic loss and cognitive impairment in an animal model of sepsis-associated encephalopathy. J Neuroinflammation.

[66] Chavan SS, Huerta PT, Robbiati S (2012). HMGB1 mediates cognitive impairment in sepsis survivors. Mol Med.

[67] Yang K, Fan M, Wang X (2022). Lactate promotes macrophage HMGB1 lactylation, acetylation, and exosomal release in polymicrobial sepsis. Cell Death Differ.

[68] Tang Y, Wang X, Li Z (2021). Heparin prevents caspase-11-dependent septic lethality independent of anticoagulant properties. Immunity.

[69] Deng M, Tang Y, Li W (2018). The Endotoxin Delivery Protein HMGB1 Mediates Caspase-11-Dependent Lethality in Sepsis. Immunity.

[70] Li SM, Li B, Zhang L (2020). A complement-microglial axis driving inhibitory synapse related protein loss might contribute to systemic inflammation-induced cognitive impairment. Int Immunopharmacol.

[71] Dejanovic B, Wu T, Tsai MC (2022). Complement C1q-dependent excitatory and inhibitory synapse elimination by astrocytes and microglia in Alzheimer's disease mouse models. Nat Aging.

[72] Coley AA, Gao WJ (2019). PSD-95 deficiency disrupts PFC-associated function and behavior during neurodevelopment. Sci Rep.

[73] Zong MM, Zhou ZQ, Ji MH, Jia M, Tang H, Yang JJ (2019). Activation of β2-Adrenoceptor Attenuates Sepsis-Induced Hippocampus-Dependent Cognitive Impairments by Reversing Neuroinflammation and Synaptic Abnormalities. Front Cell Neurosci.

[74] Moraes CA, Hottz ED, Dos Santos Ornellas D (2023). Microglial NLRP3 Inflammasome Induces Excitatory Synaptic Loss Through IL-1β-Enriched Microvesicle Release: Implications for Sepsis-Associated Encephalopathy. Mol Neurobiol.

[75] Jiang J, Zou Y, Xie C (2023). Oxytocin alleviates cognitive and memory impairments by decreasing hippocampal microglial activation and synaptic defects via OXTR/ERK/STAT3 pathway in a mouse model of sepsis-associated encephalopathy. Brain Behav Immun.

[76] Wu Y, Li P, Liu L (2022). lncRNA Neat1 regulates neuronal dysfunction post-sepsis via stabilization of hemoglobin subunit beta. Mol Ther.

[77] Figurov A, Pozzo-Miller LD, Olafsson P, Wang T, Lu B (1996). Regulation of synaptic responses to high-frequency stimulation and LTP by neurotrophins in the hippocampus. Nature.

[78] Gao LL, Wang ZH, Mu YH, Liu ZL, Pang L (2022). Emodin Promotes Autophagy and Prevents Apoptosis in Sepsis-Associated Encephalopathy through Activating BDNF/TrkB Signaling. Pathobiology.

[79] Hippensteel JA, Anderson BJ, Orfila JE (2019). Circulating heparan sulfate fragments mediate septic cognitive dysfunction. J Clin Invest.

[80] Zhang X, Han X, Xia K (2019). Circulating heparin oligosaccharides rapidly target the hippocampus in sepsis, potentially impacting cognitive functions. Proc Natl Acad Sci U S A.

[81] Girard TD, Thompson JL, Pandharipande PP (2018). Clinical phenotypes of delirium during critical illness and severity of subsequent long-term cognitive impairment: a prospective cohort study. Lancet Respir Med.

[82] Inouye SK, van Dyck CH, Alessi CA, Balkin S, Siegal AP, Horwitz RI (1990). Clarifying confusion: the confusion assessment method. A new method for detection of delirium. Ann Intern Med.

[83] Oberhaus J, Wang W, Mickle AM (2021). Evaluation of the 3-Minute Diagnostic Confusion Assessment Method for Identification of Postoperative Delirium in Older Patients. JAMA Netw Open.

[84] Lucke JA, De Gelder J, Blomaard LC (2019). CAM-ICU may not be the optimal screening tool for early delirium screening in older emergency department patients: a prospective cohort study. Eur J Emerg Med.

[85] van den Boogaard M, Pickkers P, Slooter AJ (2012). Development and validation of PRE-DELIRIC (PREdiction of DELIRium in ICu patients) delirium prediction model for intensive care patients: observational multicentre study. BMJ.

[86] Wassenaar A, van den Boogaard M, van Achterberg T (2015). Multinational development and validation of an early prediction model for delirium in ICU patients. Intensive Care Med.

[87] Cowan SL, Preller J, Goudie RJB (2020). Evaluation of the E-PRE-DELIRIC prediction model for ICU delirium: a retrospective validation in a UK general ICU. Crit Care.

[88] Jung JW, Hwang S, Ko S (2022). A machine-learning model to predict postoperative delirium following knee arthroplasty using electronic health records. BMC Psychiatry.

[89] Yadgir SR, Engstrom C, Jacobsohn GC (2022). Machine learning-assisted screening for cognitive impairment in the emergency department. J Am Geriatr Soc.

[90] Jauk S, Kramer D, Avian A, Berghold A, Leodolter W, Schulz S (2021). Technology Acceptance of a Machine Learning Algorithm Predicting Delirium in a Clinical Setting: a Mixed-Methods Study. J Med Syst.

[91] Xie Q, Wang X, Pei J (2022). Machine Learning-Based Prediction Models for Delirium: A Systematic Review and Meta-Analysis. J Am Med Dir Assoc.

[92] Wu L, Feng Q, Ai ML (2020). The dynamic change of serum S100B levels from day 1 to day 3 is more associated with sepsis-associated encephalopathy. Sci Rep.

[93] Yao B, Zhang LN, Ai YH, Liu ZY, Huang L (2014). Serum S100β is a better biomarker than neuron-specific enolase for sepsis-associated encephalopathy and determining its prognosis: a prospective and observational study. Neurochem Res.

[94] Czeiter E, Amrein K, Gravesteijn BY (2020). Blood biomarkers on admission in acute traumatic brain injury: Relations to severity, CT findings and care path in the CENTER-TBI study. EBioMedicine.

[95] Akin M, Garcheva V, Sieweke JT (2021). Neuromarkers and neurological outcome in out-of-hospital cardiac arrest patients treated with therapeutic hypothermia-experience from the HAnnover COoling REgistry (HACORE). PLoS One.

[96] Ehler J, Barrett LK, Taylor V (2017). Translational evidence for two distinct patterns of neuroaxonal injury in sepsis: a longitudinal, prospective translational study. Crit Care.

[97] Ehler J, Petzold A, Wittstock M (2019). The prognostic value of neurofilament levels in patients with sepsis-associated encephalopathy - A prospective, pilot observational study. PLoS One.

[98] Page VJ, Watne LO, Heslegrave A (2022). Plasma neurofilament light chain protein as a predictor of days in delirium and deep sedation, mortality and length of stay in critically ill patients. EBioMedicine.

[99] Nielsen RM, Urdanibia-Centelles O, Vedel-Larsen E (2020). Continuous EEG Monitoring in a Consecutive Patient Cohort with Sepsis and Delirium. Neurocrit Care.

[100] Semmler A, Widmann CN, Okulla T (2013). Persistent cognitive impairment, hippocampal atrophy and EEG changes in sepsis survivors. J Neurol Neurosurg Psychiatry.

[101] Gilmore EJ, Gaspard N, Choi HA (2015). Acute brain failure in severe sepsis: a prospective study in the medical intensive care unit utilizing continuous EEG monitoring. Intensive Care Med.

[102] de Araújo BES, da Silva Fontana R, de Magalhães-Barbosa MC (2022). Clinical features, electroencephalogram, and biomarkers in pediatric sepsis-associated encephalopathy. Sci Rep.

[103] Stubbs DJ, Yamamoto AK, Menon DK (2013). Imaging in sepsis-associated encephalopathy-insights and opportunities. Nat Rev Neurol.

[104] Heming N, Mazeraud A, Verdonk F, Bozza FA, Chrétien F, Sharshar T (2017). Neuroanatomy of sepsis-associated encephalopathy. Crit Care.

[105] Ji M, Xia J, Tang X, Yang J (2018). Altered functional connectivity within the default mode network in two animal models with opposing episodic memories. PLoS One.

[106] Zhang Y, Wu W, Toll RT (2021). Identification of psychiatric disorder subtypes from functional connectivity patterns in resting-state electroencephalography. Nat Biomed Eng.

[107] Chen HJ, Wen J, Qi R (2018). Re-Establishing Brain Networks in Patients with ESRD after Successful Kidney Transplantation. Clin J Am Soc Nephrol.

[108] Li H, Liao H, Zhang C (2022). Disrupted metabolic and spontaneous neuronal activity of hippocampus in sepsis associated encephalopathy rats: A study combining magnetic resonance spectroscopy and resting-state functional magnetic resonance imaging. Front Neurosci.

[109] Giridharan VV, Generoso JS, Lence L (2022). A crosstalk between gut and brain in sepsis-induced cognitive decline. J Neuroinflammation.

[110] Tóth M, Doorduin J, Häggkvist J (2015). Positron Emission Tomography studies with [11C]PBR28 in the Healthy Rodent Brain: Validating SUV as an Outcome Measure of Neuroinflammation. PLoS One.

[111] Xiang J, Tao Y, Xia Y (2023). Development of an α-synuclein positron emission tomography tracer for imaging synucleinopathies. Cell.

[112] Zhao Z, Wang Y, Zhou R (2020). A novel role of NLRP3-generated IL-1β in the acute-chronic transition of peripheral lipopolysaccharide-elicited neuroinflammation: implications for sepsis-associated neurodegeneration. J Neuroinflammation.

[113] Martin GS, Mannino DM, Eaton S, Moss M (2003). The epidemiology of sepsis in the United States from 1979 through 2000. N Engl J Med.

[114] Wiersinga WJ, Rhodes A, Cheng AC, Peacock SJ, Prescott HC (2020). Pathophysiology, Transmission, Diagnosis, and Treatment of Coronavirus Disease 2019 (COVID-19): A Review. JAMA.

[115] Prescott HC, Angus DC (2018). Enhancing Recovery From Sepsis: A Review. JAMA.

[116] Hill AR, Spencer-Segal JL (2021). Glucocorticoids and the Brain after Critical Illness. Endocrinology.

[117] Keh D, Trips E, Marx G (2016). Effect of Hydrocortisone on Development of Shock Among Patients With Severe Sepsis: The HYPRESS Randomized Clinical Trial. JAMA.

[118] Kawazoe Y, Miyamoto K, Morimoto T (2017). Effect of Dexmedetomidine on Mortality and Ventilator-Free Days in Patients Requiring Mechanical Ventilation With Sepsis: A Randomized Clinical Trial. JAMA.

[119] Lewis K, Piticaru J, Chaudhuri D (2021). Safety and Efficacy of Dexmedetomidine in Acutely Ill Adults Requiring Noninvasive Ventilation: A Systematic Review and Meta-analysis of Randomized Trials. Chest.

[120] Hughes CG, Mailloux PT, Devlin JW (2021). Dexmedetomidine or Propofol for Sedation in Mechanically Ventilated Adults with Sepsis. N Engl J Med.

[121] Heybati K, Zhou F, Ali S (2022). Outcomes of dexmedetomidine versus propofol sedation in critically ill adults requiring mechanical ventilation: a systematic review and meta-analysis of randomised controlled trials. Br J Anaesth.

[122] Iwashyna TJ, Cooke CR, Wunsch H, Kahn JM (2012). Population burden of long-term survivorship after severe sepsis in older Americans. J Am Geriatr Soc.

[123] Fleischmann-Struzek C, Rose N, Freytag A (2021). Epidemiology and Costs of Postsepsis Morbidity, Nursing Care Dependency, and Mortality in Germany, 2013 to 2017. JAMA Netw Open.

[124] Chao PW, Shih CJ, Lee YJ (2014). Association of postdischarge rehabilitation with mortality in intensive care unit survivors of sepsis. Am J Respir Crit Care Med.

[125] McLennan S, Shaw D, Celi LA (2019). The challenge of local consent requirements for global critical care databases. Intensive Care Med.

[126] Harhay MO, Casey JD, Clement M (2020). Contemporary strategies to improve clinical trial design for critical care research: insights from the First Critical Care Clinical Trialists Workshop. Intensive Care Med.

[127] GBD 2021 Antimicrobial Resistance Collaborators (2024). Global burden of bacterial antimicrobial resistance 1990-2021: a systematic analysis with forecasts to 2050. Lancet.

[128] Abraham E (1999). Why immunomodulatory therapies have not worked in sepsis. Intensive Care Med.

[129] Opal SM, Laterre PF, Francois B (2013). Effect of eritoran, an antagonist of MD2-TLR4, on mortality in patients with severe sepsis: the ACCESS randomized trial. JAMA.

[130] Cavaillon JM (2023). During Sepsis and COVID-19, the Pro-Inflammatory and Anti-Inflammatory Responses Are Concomitant. Clin Rev Allergy Immunol.

[131] Seemann S, Zohles F, Lupp A (2017). Comprehensive comparison of three different animal models for systemic inflammation. J Biomed Sci.

[132] De Backer D, Deutschman CS, Hellman J (2024). Surviving Sepsis Campaign Research Priorities 2023. Crit Care Med.

[133] Ely EW, Margolin R, Francis J (2001). Evaluation of delirium in critically ill patients: validation of the Confusion Assessment Method for the Intensive Care Unit (CAM-ICU). Crit Care Med.

[134] Bergeron N, Dubois MJ, Dumont M, Dial S, Skrobik Y (2001). Intensive Care Delirium Screening Checklist: evaluation of a new screening tool. Intensive Care Med.

[135] Penna GL, Fialho FM, Kurtz P (2013). Changing sedative infusion from propofol to midazolam improves sublingual microcirculatory perfusion in patients with septic shock. J Crit Care.

[136] Ohta Y, Miyamoto K, Kawazoe Y, Yamamura H, Morimoto T (2020). Effect of dexmedetomidine on inflammation in patients with sepsis requiring mechanical ventilation: a sub-analysis of a multicenter randomized clinical trial. Crit Care.

[137] Miyamoto K, Nakashima T, Shima N (2018). Effect of Dexmedetomidine on Lactate Clearance in Patients With Septic Shock: Subanalysis of a Multicenter Randomized Controlled Trial. Shock.

[138] Morelli A, Sanfilippo F, Arnemann P (2019). The Effect of Propofol and Dexmedetomidine Sedation on Norepinephrine Requirements in Septic Shock Patients: A Crossover Trial. Crit Care Med.

